# A genetic variant in microRNA-146a is associated with sporadic breast cancer in a Southern Brazilian Population

**DOI:** 10.1590/1678-4685-GMB-2019-0278

**Published:** 2020-02-03

**Authors:** Heloisa Magagnin Brincas, Danillo G. Augusto, Carolina Mathias, Iglenir João Cavalli, Rubens Silveira de Lima, Flávia Kuroda, Cícero de Andrade Urban, Daniela Fiori Gradia, Jaqueline de Oliveira, Rodrigo Coutinho de Almeida, Enilze Maria de Souza Fonseca Ribeiro

**Affiliations:** 1 Universidade Federal do Paraná, Departamento de Genética, Curitiba, Paraná, Brazil.; 2 Hospital Nossa Senhora das Graças, Centro de Doenças da Mama, Curitiba, Paraná, Brazil.

**Keywords:** miRNA polymorphism, miR146a, rs2910164, Breast cancer, Case-control study

## Abstract

MicroRNAs (miRNAs) play an essential role in gene expression and affect the development of tumours, including breast cancer (BC). Polymorphisms in miRNA genes can affect the interaction of miRNAs with their target messenger RNA by interfering, creating or disrupting target sites. The single nucleotide polymorphism (SNP) *rs2910164*, located in the seed region of miR146a, was shown to be associated with BC among different populations. In the present study, we investigated whether *rs2910164* is associated with BC in 326 patients and 411 controls from a Brazilian population of predominantly European ancestry. The presence of the allele rs2910164*C was associated with an increased risk of BC (OR=1.4, 95% CI=1.03-1.85, *p* = 0.03). We also analysed publicly available RNA-seq data to evaluate if miR146a is differentially expressed in different subtypes of BC. Genotyping was performed by polymerase chain reaction with sequence-specific primers (PCR-SSP). By leveraging public data from TCGA database, we analysed 461 patients and found that miR146a is significantly more expressed in BC than in non-tumor tissue (1.47 fold, *p* = 0.02) and is expressed to a greater degree in aggressive BC subtypes.

## Introduction

Breast cancer (BC) is the second most frequent type of cancer in Brazilian women (INCA, 2018) and is considered the leading cause of death by malignant disease among women worldwide (Bray *et al.*, 2018). Identifying genetic factors associated with BC can aid in predicting disease risk at the individual and population levels, and provide information about the underlying disease mechanism. Some of the genetic variants implicated in BC are SNPs (single nucleotide polymorphisms) located in microRNA (miRNA) genes (Alshatwi *et al.*, 2012).

miRNAs are a highly conserved class of small (~22 nucleotide) endogenous non-coding RNAs (Bartel, 2004). They play an essential role in cancer development by modulating the expression of oncogenes and tumour suppressor genes (Croce, 2009). Small alterations in the miRNA sequence can lead to substantial phenotypic consequences. Their function can be modified in three main ways: 1) altered transcription levels of the primary transcript; 2) altered processing and transport of the pri-miRNA and pre-miRNA; 3) altered interaction of the miRNA with their target mRNAs (Ryan *et al.*, 2010).

Genome-wide association studies (GWAS) previously identified 102 loci associated with BC in Europeans (Michailidou *et al.*, 2017). GWAS showed that 90% of the SNPs associated with complex diseases were located in non-protein coding regions, including the ones transcribed to miRNAs (Hrdlickova *et al.*, 2014; Almeida *et al.*, 2018). Notwithstanding, SNPs have been found in only 10% of the known pre-miRNAs. Within the seed region, Variation occurs in only less than 1% of the known seed regions, which consists of the nucleotides 2-8 from the mature miRNA sequence. The seed region specifies to which mRNAs the particular miRNA will be able to bind and the lack of variation suggests a purifying selection (Saunders *et al.*, 2007).

The *mir146a* gene is located at the chromosomal region 5q33.3 and encodes the miR146a-3p and the miR146a-5p. The SNP *rs2910164* is a *G* > *C* alteration at position +60 from the first nucleotide of the pre-miR146a (Jazdzewski *et al.*, 2008), on the seed region of miR146a-3p. The result is a G:U to C:U change leading to a weaker pairing within the pre-miRNA hairpin structure (Hu *et al.*, 2008), which alters the processing efficiency of pre-miR146a. It has been shown that the amount of mature miR146a is two times lower with the *C* allele. This difference is due to a less efficient pri-miRNA processing and a reduced transport of pre-miR146a from the nucleus to the cytoplasm (Jazdzewski *et al.*, 2008).

There are few case-controls studies focusing the polymorphism *rs2910164* in association with BC. Five studies were described in the European population (Catucci *et al.*, 2010; Pastrello *et al.*, 2010; Garcia *et al.*, 2011; Cardeñosa *et al.*, 2012; McVeigh *et al.*, 2017) and one in an Australian Caucasian population (Uphadhyaya *et al.*, 2015). The majority of these studies have focused on familial BC (Catucci *et al.*, 2010; Pastrello *et al.*, 2010; Garcia *et al.*, 2011; Cardeñosa *et al.*, 2012). The results are conflicting showing positive association in only two studies, with *C* allele (*GC*+*CC*) in Pastrello *et al.* (2010) and with *G* allele in sporadic BC in Uphadhyaya *et al.* (2015).

Comparing patients with triple-negative breast cancer (TNBC) and no TNBC, we recently demonstrated that miR146a-5p presents a higher level of expression in TNBC (Sugita *et al.*, 2016). This result motivated the investigation of whether *rs2910164* is associated with BC in a Southern Brazilian population and to evaluate the expression of miR146a-5p among subtypes of BC.

## Subjects and Methods

### Study cohort

The study population consisted of 326 Brazilian women diagnosed with sporadic BC, from the Nossa Senhora das Graças Hospital, Curitiba, Brazil. The control group comprised 411 healthy Brazilian women from the bone marrow donor biobank of the Laboratory of Immunogenetics and Histocompatibility, Genetics Department, Federal University of Paraná, Curitiba. Both patients and controls were from the same region in South Brazil and belong to the same ethnic background, which is of predominantly European ancestry (Probst *et al.*, 2000; Sugita *et al.*, 2016). This study was approved by the Brazilian Commission of Ethics in Research (CONEP) under the number CAAE 67400917.3.0000.55 following Brazilian Federal laws. All participants signed informed written consent following the principles of the Declaration of Helsinki. The mean ages of the case and the control groups were 56.23 ± 15 and 47.66 ± 4.69, respectively. Histopathological parameters, immunohistochemical features, and status of regional lymph node invasion from cancer samples are summarized in Table 1. The immunohistochemical classification was based on Goldhirsch *et al.* (2013).

**Table 1:** Histopathological and immunohistochemical parameters of breast cancer patients.

Breast cancer patients n=326					
**Histology of carcinoma**	**n**	**%**		**Tumour Grade**	**n**	**%**
Invasive ductal	233	75%		Grade I	37	16%
Invasive lobular	28	9%		Grade II	125	53%
Ductal *in situ*	12	4%		Grade III	75	32%
Invasive mucinous	9	3%		Without Information	89	-
Others	28	9%				
Without information	16	-				
**Immunohistochemical classification**	**n**	**%**		**Lymph node invasion**	**n**	**%**
Luminal A	84	40%		Present	116	47%
Luminal B	78	38%		Absent	133	53%
HER2+	15	7%		Without information	77	-
Triple-Negative	31	15%				
Without information	118	-				

### Genotyping

The SNP *rs2910164* was genotyped using polymerase chain reaction with specific sequence primers (PCR-SSP). The primer sequences used were: forward 5’-GGTTGTGTCAGTGTCAGACCTC-3’, forward 5’-GGTTGTGTCAGTGTCAGACCTG-3’, reverse 5’-GAGCCTGAGACTCTGCCTTCT-3’ and the product size was 196 bp. PCR amplifications were carried out in a 20 μL reaction containing: 40 ng DNA, 0.2 μM each primer, 0.2mM dNTPs, KCl 50 mM, Tris-HCl 10 mM and 1.25 U Taq polymerase (Invitrogen, Carlsbad, CA, USA). PCR conditions were: 95 °C for 1 min, followed by 35 cycles of 95 °C for 30 s, 59 °C for 30 s and 72 °C for 30 s, followed by 5 min at 72 °C. After the reaction, the products were analysed by gel electrophoresis in 2% agarose gel stained with GelRed® (Biotium, Fremont, CA, USA). As an internal amplification control, we amplified the *galactosylceramidase* gene (*GALC*) using the following primers: forward 5’- TTACCCAGAGCCCTATCGTTCT -3’ and reverse 5’- GTCTGCCCATCACCACCTATT -3’. The *GALC* amplicon (352 bp) was expected in all reactions. We validated the specificity and accuracy of our PCR-SSP method by sequencing five random individuals with Sanger methods and verifying their genotypes.

### Expression analysis from publicly available RNA-Seq data

The Cancer Genome Atlas Program (TCGA) is a public database that contains data from thousands of matched cancer and non-cancer samples (TCGA Network, 2012). We analysed RNA-Seq data from 837 BC patients and 105 adjacent tissues to search for possible differential expression levels of miR146a in BC compared to non-tumour tissue and among intrinsic subtypes. Pre-processing and normalization of RNA-seq data were performed by TCGA biolinks package from R/Bioconductor (Colaprico *et al.*, 2016). For expression analysis from molecular subtypes, we analysed 461 patients, being 211 Luminal A, 112 Luminal B, 85 Basal-like, and 53 HER2-Enriched.

### Statistical analysis

Association analysis was performed using chi-square test of independence and using odds ratios (OR) and 95% confidence intervals (Mantel and Haenszel, 1959). We used R package Hardy-Weinberg (Graffelman, 2015) to test if genotype distributions were under Hardy Weinberg equilibrium. A chi-square test of independence was used to compare the distribution of *rs2910164* genotypes among the several parameters (histopathological, subgroups defined by immunohistochemistry, tumour grade, and lymph node invasion). Bartlett’s test was applied to compare the age of cancer diagnosis in different genotypes (Bartlett, 1937). Analysis of the expression data from TCGA was performed using Kruskal-Wallis test followed by Dunn test (Kruskal and Wallis, 1952; Dunn, 1964). Spearman correlation was used to check the correlation of miR146a and the genes *BRCA1*, *TRAF6*, *IRAK1*, *TRAF3IP1*.

Alpha (α) was set at 0.05 for all tests and all analyses were performed with R Software (R core team, 2017) with the packages Nortest and readxl (Gross and Ligges, 2015; Wickham and Bryan, 2017).[Bibr B46]

## Results

The presence of the allele rs2910164C is associated with breast cancer risk

The genotype *GC* was more frequent in patients compared to controls (Table 2). Considering that the difference in the distribution of the three genotypes was not significant (*p* = 0.10), we determined the magnitude of the effect on BC susceptibility testing three models by odds ratio tests: *G* vs. *C* for additive, *CC* + *GC* vs. *GG* for dominant and *CC* vs. *GC* + *GG* for recessive inheritance. The dominant model (presence of allele *C*) was associated with an increased risk of BC (OR = 1.4, *p* = 0.03). The *C* allele was observed 4% more frequently in the case than in the control population. The distribution of genotypes followed Hardy-Weinberg equilibrium in the control population (*p* = 0.70).

**Table 2:** Genotypic and allelic frequencies of the SNP *rs2910164* in breast cancer patients and controls.

	Patients (n = 326)	Controls (n = 411)			
**Allele**	***f***	***f***	**OR**	***p***	**95%CI**
***G***	0.730	0.769	ns		
***C***	0.270	0.231	ns		
**Genotype**	**% (n)**	**% (n)**			
***G+***	95.1 (310)	94.9 (390)	ns		
***C+***	49.1 (160)	41.1 (169)	1.4	0.03	1.03 - 1.85
***GG***	50.9 (166)	58.9 (242)	ns		
***GC***	44.2 (144)	36 (148)	1.4	0.03	1.04 - 1.89
***CC***	4.9 (16)	5.1 (21)	ns		

Legend: *G+* = carriers of allele *G* (*GG*+*GC*), *C+* = carriers of allele *C* (*CC*+*GC*), *f* =allelic frequency, OR = odds ratio; *p* = *p*-value; 95% CI = 95% confidence interval; ns = not significant.

We observed no differences in distribution of lymph node invasion (*p* = 0.25), tumour grade (*p* = 0.30) and immunohistochemical subgroups (*p* = 0.90) comparing risk group *GC*+*CC* vs. *GG* group from BC patients. Applying Bartlett test for homogeneity, no difference was observed in the variance analysis for assessing the risk of developing BC in younger ages for the risk group *GC*+*CC* vs. the *GG* group.

### miR146a is differentially expressed in breast cancer

**Figure 1:**
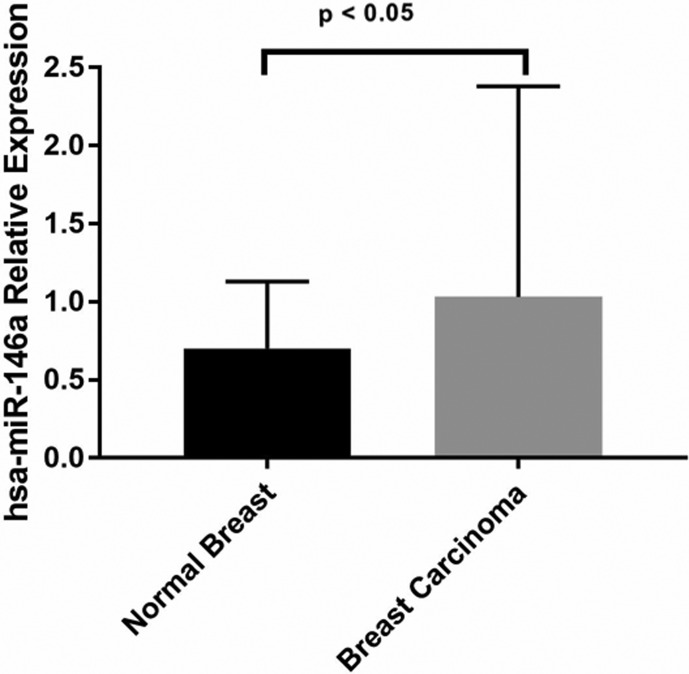
Higher expression levels of hsa-miR-146a in breast cancer analysis from TCGA RNA-seq data

**Figure 2:**
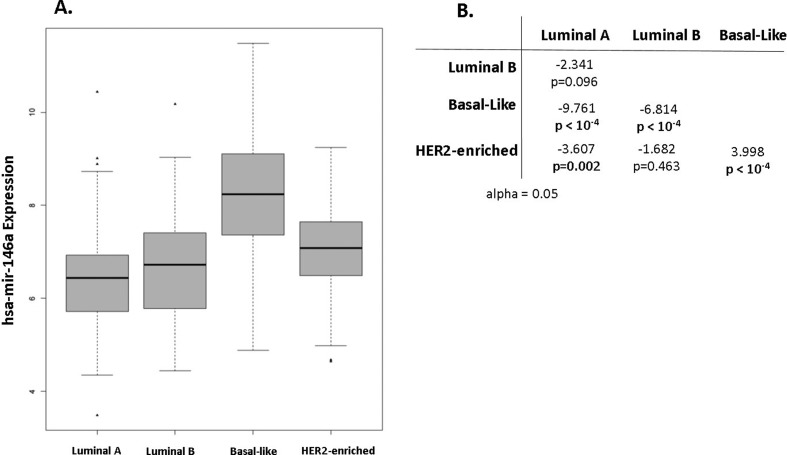
A. Comparison of hsa-mir-146a expression in breast cancer intrinsic subtypes; B. Dunn’s test results summarized.

Accessing publicly available RNA-Seq data from TCGA database, we found miR146a significantly more expressed in tumour tissue compared to non-tumour adjacent tissue (Figure 1). Kruskal-Wallis analysis and Dunn’s test indicated significant differences in the expression of miR146a between each molecular subtype (Figure 2).

Spearman correlation analysis was performed to analyse the expression of miR146a-5p and predicted target genes, *BRCA1*, *IRAK1, TRAF3IP1*, and *TRAF6* using TCGA RNA-seq data. No correlation with *BRCA1* was found (rho= -0.02, *p*=0.58) However, we observed a negative correlation between miR146a-5p expression levels with *TRAF6* (rho= -0.13, *p*= 0.0002) and *TRAF3IP1* (rho=-0.27, *p*= 8.5e-14), and a positive correlation of miR146a-5p with *IRAK1* expression (rho= 0.27, *p*= 3.5x10^-14^). For *BRCA1* we tested the correlation with miR146a-5p within each molecular subtype but did not find correlation in any of them. Also, *rs2910164* was not found in linkage disequilibrium with any other variant described at ENSEMBL database and 1000genomes database in any population (The 1000 Genomes Project Consortium, 2015; Zerbino *et al.*, 2018).

## Discussion

The allelic frequencies of *rs2910164* vary between populations; as a result, some alleles that may confer risk in a particular population may be the same that are protective in others (Upadhyaya *et al.*, 2016). The allelic frequencies of *rs2910164* observed in the control group of the present study were similar to the Europeans from Hapmap (CEU G-76% and C-24%) (International HapMap Consortium, 2003), as well as the Europeans from NHLBI Exome Sequencing Project (NHLBI E_A G-77% and C-23%) (The 1000 Genomes Project Consortium, 2015). This result is following the previously reported predominantly European ancestry of the population from Southern Brazil (Probst *et al.*, 2000; Sugita *et al.*, 2016).

The association of *rs2910164* and BC was studied in several studies from different populations (Xu *et al.*, 2013; Dai *et al.*, 2015; Uphadhyaya *et al.*, 2015; Barjui *et al.*, 2017; Zhang *et al.*, 2017). Dai *et al.* (2015) showed that the effect of *rs2910164* on BC susceptibility is different across populations. The authors suggest that the allele *C* is associated with BC risk in Europeans (2777 cases and 2898 controls) but not in Asians (1537 cases and 1587 controls). This discrepancy could be explained by the interaction of *rs2910164* with different genetic backgrounds, by different exposure to carcinogens, or due to linkage disequilibrium with a different causal variant. However, we have not found significant LD between *rs2910164* with any other polymorphism described in either ENSEMBL or the 1000genomes database. Our results, therefore, suggest that LD is not the primary factor influencing the different associations observed for *rs2910164* with BC. Also evidencing the differences of the effect of *rs2910164* on the risk of BC in diverse populations, the allele *rs2910164G* was associated with increased risk of sporadic BC in Australians (OR =1.77, p < 10^-4^; Uphadhyaya *et al.*, 2015) and Iranians (OR = 1.91*, p* = 0.03; Barjui *et al.*, 2017). Other studies, on the other hand, reported no association (Zhang *et al.*, 2016; Mu *et al.*, 2017).

We observed a higher frequency of patients carrying the allele *rs2910164C* in comparison to controls. Although the rates of the genotype *CC* were similar in both groups, this genotype was observed in relatively low frequency (5%). It is plausible to expect that studying larger cohorts could reveal differences between rates of the genotype *CC* in cases and controls. In fact, increased risk for *CC* genotype was observed by different studies that analysed Europeans (Lian *et al.*, 2012; Dai *et al.*, 2015).

Younger age at the time of diagnosis is an important prognostic marker for BC, also associated with hereditary cases (Armaou *et al.*, 2009). In a study involving 40 American women with hereditary BC, the presence of the allele *rs2910164C* was increased in women diagnosed at younger ages, with a median age of 45 versus 56 (*p*=0.029; Shen *et al.*, 2008). A study of 348 Italian women with hereditary BC showed similar results with a median age at diagnosis of 35 versus 52; (*p*=0.028; Pastrello *et al.*, 2010). On the other hand, a third study reported a lack of association with younger age (Catucci *et al.*, 2010). Studying sporadic BC cases, we did not find any association of *rs2910164* with the age of diagnosis. These findings were similar to those observed in other studies with sporadic BC, and this possible difference in the association between sporadic and hereditary BC with *rs2910164* is still not understood (Cardeñosa *et al.*, 2012; Qi *et al.*, 2015; Barjui *et al.*, 2017; Mcveigh *et al.*, 2017).

We also did not find any association between genotypes of *rs2910164* and regional lymph node invasion, immunohistochemical subgroups or tumour grade, which suggests a lack of association of *rs2910164* with tumour progression. This result is similar to studies in the Irish population (McVeigh *et al.*, 2017) and the Chinese (Qi *et al.*, 2015).

We found that miR146a was significantly more expressed in BC than in healthy tissue. The highest expression was observed in basal-like tumours, which have partial overlap with TNBC in immunohistochemical classification. Among the BC subgroups defined by immunohistochemistry, triple-negative is recognized to inactivate *BRCA1* tumour suppressor gene (Turner *et al.*, 2007). A reduced expression level of *BRCA1* is observed in a significant proportion of sporadic BC cases, despite the absence of somatic mutations in *BRCA1* (Mueller and Roskelley, 2003). Interestingly, miR146a-5p was predicted to target *BRCA1* and thereby reduce its expression (Shen *et al.*, 2008; Garcia *et al.*, 2011). Also, the expression levels of miR146a-5p were observed three times higher in triple-negative tumours compared to other subgroups of mammary tumours (M’Hamed *et al.*, 2015). The hypothesis of regulation of *BRCA1* expression by miR146a-5p was not corroborated in our analysis using RNA-Seq data. This analysis did not show a correlation between expression levels of miR146a and *BRCA1* in BC in general, neither in basal-like patients.

Studies suggest that *TRAF6*, *IRAK1* (Bhaumik *et al.*, 2008) and *EGFR*, a protein associated with tumour progression and metastasis (Kumaraswamy *et al.*, 2016), are targets for mir146a-5p. Our analysis showed a negative correlation between the expression of mir146a-5p with *TRAF6* as well as with *TRAF3IP1*. However, we found a positive correlation of miR146a-5p with *IRAK1*. It has been shown that reduced *TRAF6* and *IRAK1* levels lessen the activity of NF-kB, a potential inducer of proliferation, survival, angiogenesis, and metastasis (Bhaumik *et al.*, 2008). In summary, the studies on miR146a and their targets suggest that *rs2910164* can exert their effects on cancer mainly by changing the binding to the target rather than by modulating the expression levels of miR146a-5p. However, further studies are needed to validate these targets and to provide direct evidence of the regulation by miR146a.

In conclusion, our study provides insights into the role of miR146a in a Brazilian population of predominantly European ancestry. We show the presence of *rs2910164C* increasing the susceptibility to sporadic BC among women despite its apparent lack of influence in disease progression. We observed significantly higher expression levels of miR146a-5p associated with the severity of BC, the highest expression observed being in patients with basal-like tumours. Our results point to the importance of miRNAs in the susceptibility to cancer and suggest that functional studies will further elucidate the impact of miR146a in breast cancer.
